# Supplementary material for: SyNDI: synchronous network data integration framework
Source: BMC Bioinformatics. 2018 Nov 6;19:403. doi: 10.1186/s12859-018-2426-5 (PMC6219086; doi:10.1186/s12859-018-2426-5)
Supplement: Supplementary file 5 — Files of the example presented in Additional file 4. This additional file is a zip package that contains all files used in Additional file 4. Detailed descriptions of each of these files are presented in a README file included in this zip package. (GZ 30411 kb) [file 12859_2018_2426_MOESM5_ESM.gz › SyNDITools/Meme2Fimo/meme_4.9.0_4.tar.gz!/meme_4.9.0/doc/examples/glam2scan_example_output_files.tgz!/glam2scan_example_output_files/glam2scan.html]

GLAM2SCAN

# GLAM2SCAN

Version 1056

```
 /home/tbailey/trunk/INSTALLED/bin/glam2scan -n 25 p aln /home/tbailey/trunk/INSTALLED/db/fasta_databases/c_elegans.aa
```

If you use this program in your research, please cite: MC Frith,
NFW Saunders, B Kobe, TL Bailey (2008) Discovering sequence motifs with
arbitrary insertions and deletions, PLoS Computational Biology, **4**(5), e1000071, 2008.

| NAME | START | SITE | END | STRAND | SCORE |
| --- | --- | --- | --- | --- | --- |
|  |  |  |  |  |  |
| --- | --- | --- | --- | --- | --- |
| F36A2.1a | 49 | NCFK.TAGTDNKRIALFYVMN | 68 | + | 19.0 |
| F36A2.1b | 49 | NCFK.TAGTDNKRIALFYVMN | 68 | + | 19.0 |
| M01A10.1 | 631 | NKAR.IMETDYSAQWLQYYTQ | 650 | + | 18.5 |
| T06E6.7 | 172 | NMKLFVLATDYNLFVVELVFM | 192 | + | 18.2 |
| F41G3.12 | 326 | NSAH.VCGTDGKTYLNECFLK | 345 | + | 18.0 |
| C38H2.2 | 177 | NDYDWFLKADDDTYVVMENLR | 197 | + | 17.8 |
| Y59H11AR.3 | 88 | YWIK.VLQTDYEISFLIKHNCT | 108 | + | 17.8 |
| T20F10.4 | 130 | NTFGWILETKDIMTYKINKYTK | 151 | + | 17.5 |
| Y26D4A.10 | 130 | NTFGWILETKDIMTYKINKYTK | 151 | + | 17.5 |
| Y40C7B.3 | 130 | NTFGWILETKDIMTYKINKYTK | 151 | + | 17.5 |
| T08G3.3 | 184 | NPRLFVMGTDNNTF.IACVVP | 203 | + | 17.1 |
| C30G12.7 | 102 | NVGH.VLSTINNNY.MNHQYN | 120 | + | 16.7 |
| R13A5.7 | 54 | NIIF.IEQTDYFRNLAKYICR | 73 | + | 16.6 |
| K07C5.2 | 104 | NSLA.TLQTDYVDLLLIHYPR | 123 | + | 16.1 |
| C28A5.2 | 1192 | NTTY.IKKTDAVKMMLHYMYR | 1211 | + | 15.8 |
| ZK1225.1 | 230 | SVVP.VVGTDHSNFAVLHPEL | 249 | + | 15.7 |
| Y26D4A.2 | 286 | NFYN.VKTTD.KYYVVNTYVN | 304 | + | 15.3 |
| F36G9.1 | 6 | NEMEYYDDTDYEDFIFWNNIM | 26 | + | 15.3 |
| M03A8.1 | 206 | NLVD.ALKPDYVTPLVTYMVH | 225 | + | 15.2 |
| E03H4.3 | 155 | NSFDWYLKADDDTYFAMDHLRE | 176 | + | 14.6 |
| T24D5.1 | 163 | NHAPEIPRKDQDGYLLECNLN | 183 | + | 14.6 |
| M01A10.2c | 640 | NDDTLIYNTDTGNYTLRSTRN | 660 | + | 14.4 |
| M01A10.2d | 487 | NDDTLIYNTDTGNYTLRSTRN | 507 | + | 14.4 |
| R08D7.8 | 47 | NDCC.VTLTDYDDNVLNLLEE | 66 | + | 14.3 |
| C28A5.1 | 1197 | NTTY.IKKTDAVTMMLQFMYR | 1216 | + | 14.2 |
